# Challenges in linking health research to policy: a commentary on developing a multi-stakeholder response to orphans and vulnerable children in Ghana

**DOI:** 10.1186/1478-4505-9-S1-S14

**Published:** 2011-06-16

**Authors:** John Owusu Gyapong, Richmond Ato Selby, Kwadwo Antwi Anakwah

**Affiliations:** 1 Research and Development Division, Ghana Health Service, P O Box MB-190, Accra, Ghana

## Abstract

The Research and Development Division (RDD) of the Ghana Health Service (GHS) has a remit to build research capacity and conduct policy relevant research. By being situated within the GHS, RDD has good access to directors and programme managers, within and beyond the Ministry of Health. This structure has been facilitating collaboration through research cycles for 20 years, from agenda setting to discussions on policy relevance.

This approach has been applied to research activities within the Addressing the Balance of Burden in AIDS (ABBA) Research Programme Consortium to tackle the challenges facing HIV affected orphans and vulnerable children (OVCs). The government strategy on OVCs recommends they should be encouraged to live in their home communities rather than in institutions. We present lessons here on efforts to use research to build a response across different agencies to address the problems that communities and families face in caring for these children in their communities.

This approach to building consensus on research priorities points to the value of collaboration and dialogue with multiple stakeholders as a means of fostering ownership of a research process and supporting the relevance of research to different groups. Our experience has shown that if the context within which researchers, policy makers and stakeholders work were better understood, the links between them were improved and research were communicated more effectively, then better policy making which links across different sectors may follow. At the same time, collaboration among these different stakeholders to ensure that research meets social needs, must also satisfy the requirements of scientific rigour.

## Introduction

In this paper we share our experience of researching orphans and vulnerable children (OVC) to illustrate how the Research and Development Division (RDD) in Ghana has used an intersectoral approach to engagement with HIV policy actors. The Research and Development Division has a unique position as a research agency being located within the Ghana Health Service of the Ghana Ministry of Health; it was set up in 1990 to provide evidence for policy formulation and support for programme implementation because at that time academic research in Ghana was not perceived to be meeting the needs of the health care delivery system. Since inception of the RDD the close involvement of policy makers and programme persons in research has become more prominent. The RDD has the following functions:

•	Undertaking research in support of programme delivery and policy development and the commissioning of research to appropriate institutions;

•	Assisting the Ghana Health Service in setting health priorities through generation of new knowledge and systematic review of existing information;

•	Assisting the health sector in developing research proposals and conducting training in health systems research;

•	Serving as a documentation centre for Health Research;

•	Facilitating technical and ethical review of research

The principles guiding the operations of the RDD-GHS are that the research agenda should be set through a consultative process; it should answer policy and programme questions and be relevant to the health sector programme of work; human resource and infrastructure development should be an integral component of any research project to ensure capacity development; technical assistance for any collaborative research should be demand driven; and the highest ethical standard should be maintained.

Addressing the Balance of Burden in AIDS (ABBA) is a DFID funded project aiming to improve HIV/AIDS policy making in Africa http://www.abbarpc.org. It is a Research Programme Consortium (RPC) consisting of collaborating institutions from Kenya, Malawi, South Africa, the United Kingdom and Ghana. RDD is the Ghanaian partner of the ABBA RPC. A central objective of the ABBA partnerships is to influence poverty reduction and the achievement of the Millennium Development Goals by tackling and reducing the causes of vulnerability through improving HIV/AIDS policy-making.

One of the issues the Ghana team has addressed under the ABBA RPC is developing appropriate responses to orphaned and vulnerable children affected by HIV/AIDS. This case-study illustrates the importance of an approach which engages multiple official stakeholders in collaborating to facilitate the uptake of research evidence. Children affected by HIV in Ghana frequently live in poverty, are stigmatized by society, have poor school attendance and fail to receive the government protection and support they are entitled to. Ghana is believed to have approximately 17,500 children living with HIV infection [1]. The estimate of the number of AIDS orphans in Ghana is expected to double, increasing from 132,000 in 2004 to 291,000 in 2015 [2]. Traditionally orphans in Ghana would be absorbed in to extended families, the changing demographic profile and the projected orphan burden suggest this will become less feasible and AIDS orphans will become increasingly vulnerable unless supportive interventions reach them effectively [3]. Attempts have therefore been made by government bodies to improve the support services of OVCs, and to provide indirectly support via funding for associations working with People Living with HIV/AIDS (PLHA).

Responsibility for HIV affected OVC lies across several government departments and designing a strategy for their support has therefore necessitated a collaborative approach. The relevant agencies working with OVC are the Department of Social Welfare, the Ministry for Women and Children’s Affairs, and the Ministry of Manpower Youth and Employment. Evidence from Ghana has suggested that OVC are best supported and cared for within their extended families, rather than institutions, and this policy approach is therefore promoted (4). Additionally such an approach is generally more culturally acceptable within the Ghanaian value system than institutional care for OVC. These departments encourage childcare within the community via a range of initiatives relating to financial support, health and education targeted at HIV/AIDS OVC with additional support provided by non-governmental organizations. The Ghana AIDS Commission is an intersectoral government body with the duty to coordinate, monitor and evaluate the national response to HIV/AIDS, it is also involved in resource mobilization for HIV/AIDS OVC. The commission reports directly to the office of the president and is therefore able to involve all sector of government.

In 2007 the RDD hosted a set of consultative stakeholder meetings including policymakers, people living with HIV/AIDS, and orphanage managers at national and regional levels. The aim of the meetings was to agree on research priorities in the field of HIV and AIDS to take forward within the ABBA research programme, which has the broad remit of understanding and addressing social issues related to HIV. Following a consensus building process the discussions at the workshop highlighted gaps in understanding on OVCs in Ghana and in particular the lack of knowledge and awareness of policy guidelines for OVCs; the need to better identify OVCs in communities and consider changes to how interventions are implemented; and build awareness of organisations and services provided for OVCs.

As a result of the consultative meetings the RDD undertook a research study (2008-10) to assess the state of HIV affected OVC in households and orphanages. The study questions arose from the consultative meetings, RDD developed the study design and stakeholders were able to comment on cultural sensitivities, ethics and community entry. The objective was to describe the interventions that are available for children in different settings and to recognise the differences and commonalities between those OVCs living in households and orphanages receiving and not receiving formal support. The aim was to develop the research project with all known stakeholders involved with OVC support and work with them to disseminate the results so that a collaborative response could be developed. RDD worked closely from the project’s inception with multiple stakeholders. The stakeholders can be broadly classified into i) policy makers and practitioners, comprising the Ghana AIDS Commission (GAC), the National AIDS Control Programme, the Department of Social Welfare (DSW) and the Ministry of Women and Children’s Affairs (MOWAC); ii) academia and researchers, predominantly research scientists working on HIV/AIDS; and iii) the community, usually represented by NGOs providing HIV/AIDS services at the community level and associations of people living with AIDS.

This was a mixed-method study of a purposively selected non-randomized population. In-depth interviews were conducted with key personnel from 11 governmental and non-governmental organizations which provide services for HIV and AIDS OVC in the Central region of Ghana. In-depth interviews were also conducted with 3 caretakers of orphanages and 19 household heads that have HIV/AIDS OVC in their care [3]. Most of the in-depth interviews were more like group discussions because they required responses from different people in the orphanages. Quantitative data on the socio-demographics of households and the state (health, education and nutrition) of a sample of 137 OVC were also collected.

The government strategy has been to encourage HIV affected children to live within their extended families, however, no comprehensive database or tracking system was in place for HIV affected children, and there was limited understanding on the experiences and challenges faced by OVC and their carers in the home setting. A cash incentive system to encourage registration of OVC at a government facility had been in place in order to identify ‘needy’ children, but was associated with fraudulent activity and individual cases recorded in the database could not be validated. In addition anecdotal evidence suggested that pride prevented some families living in extreme poverty from acknowledging they needed state support; the most frequent source of external support for households interviewed in the study was through PLHA associations which receive funding from the Ghana AIDS Commission, [3].

The study found that all OVC in orphanages appeared to have an advantage in terms of education, nutrition and health care over children living in the community because they could be easily targeted for support interventions. Educational opportunities for those living in orphanages were good, with 100% of children attending school as opposed to approximately 89% OVC living in households; however children from both groups tended to be behind their age-peer group at school. OVCs in both households and orphanages showed that most were under weight, yet children in orphanages benefitted from free access to the National Health Insurance Scheme whereas children in households tended to lack the funds for health insurance. The study did not however collect data pertaining to child access to anti-retroviral treatment. Limited data regarding children living in households who had been identified for government support suggested they did receive advantages of free health and education services [3].

Preliminary findings were presented to all stakeholders and given an opportunity to ask questions and input into the process. At this stage, the main dissemination was a formality because the key stakeholders had been part of the process and were already aware of the key findings of the research project. The dissemination forum therefore focuses more on the possible actionable points and *who* should do *what* rather than just telling the story of the results. This approach makes policy makers and end users of research outcomes pay attention and become more involved in the research process.

## Discussion

From the outset, RDD set the research agenda in collaboration with policy makers and stakeholders with whom contact was maintained through facilitative intersectoral discussions and forums up until the reporting stage. The setting of the research agenda took the form of a national and regional dialogue which assembled various actors involved in policy making and program implementation in the national response to HIV/AIDS. Beyond the agenda setting stage, contact with stakeholders was active and maintained through bi-monthly face-to-face and email updates on the process. The project concluded that to make an effective and sustainable shift towards the approach of keeping more OVC in households, structures and mechanisms should be instituted to identify these children, their households and family members and provide them with the necessary support [3]

The research evidence was reported with recommendations to refocus on an intersectoral collaborative response including:

1. The DSW and MOWAC should identify and register community based OVC

2. Registered children should be supported in accessing free health care and the Free Compulsory Universal Basic Education system

3. The Ghana AIDS Commission, MOWAC and other stakeholders should develop simple educational tools to educate service providers and caregivers about the policies, support and social protection aimed at OVC

Long-term engagement with stakeholders can be complex and frustrating and this project faced several challenges. There were differences in opinion with regards prioritization of issues for the research agenda, partly due to the range of individuals participating in the process of developing the response. In the context of HIV in Ghana we face the challenge that in some policy arenas evidence from applied research is considered inferior to ‘hard science’ by officials from biomedical backgrounds. It also became apparent that the potential for stakeholders to act on recommendations was limited by considerable resource constraints. Stakeholders recognised that recommendations in the report would have a stronger chance of being implemented if the government departments and organisations could work collaboratively and communities could be empowered to access services provided.

The position of the RDD as a research body within a government body (the Ghana Health Service) had important implications for the scope of the research evidence and the outcomes from the project. Not only has RDD been able to criticise the establishment in a way that could not have been possible from a non-governmental organization, but it has also been able to use its network of links across the public sector to facilitate change. For example the Department of Social Welfare (DSW), the department with the greatest mandate to support OVCs was powerless to undertake its responsibilities without external intervention due to financial constraints. Prior to the project there had been no links between RDD and the DSW, yet RDD was able to use its links to enlist advocates to lobby the Minister for Social Welfare and thus empower the Department to carry out its work. The case of OVC was put forward to the Minister as an example of the crises within the Department, following which more resources were allocated to the department.

Based on the research engagement, in 2010 a documentary film was commissioned by RDD with the intention of disseminating key findings to a wider audience and spreading awareness of the plight of OVCs using easily an understandable format and language. This was a response to stakeholders’ suggestion that dialogue and wider recognition of the issues was necessary and social empowerment was an important strategy. The film was a ‘docu-drama’ that told the emotional story of an orphaned child living in the community, and featured interviews with high-ranking government officials. The message is exemplified in the statement from the film, “We must pull resources together, we must work in collaboration together, we must complement each other’s efforts, and once we are able to do that in harmony I think we stand a better chance to improve upon the situation with our OVC” (Ruth Addison, Head of Programmes, Ministry or Women and Children’s Affairs). The RDD links to government were such that it could negotiate that the film was aired at low cost on primetime television across the three national networks over the Easter holiday weekend. The public’s response to the documentary was striking, and prompted widespread discussion and demands for the government to act. As a response to the RDD research, the Ghana AIDS Commission expressed commitment to creating an index of OVC in Ghana to assess what services are being delivered. The links facilitated by RDD in these examples highlight the potential of intersectoral working within HIV activities.

## Conclusion

The OVC research project has produced several transferable lessons with which we can further our understanding of processes that support take up of research findings in relation to HIV and AIDS. First, continuous stakeholder engagement was essential for the effective translation and dissemination of research evidence. Second, the complexities of HIV in the community context necessitated transactional, dynamic approaches of applied research rather than a traditional scientific linear approach. Third, despite involving all relevant sectors and policy actors in the research process, translating research evidence into practice has not yet been possible due to institutional resource constraints, however engagement with policy actors allowed these limitations to be identified and action taken to rectify them. Fourth, intersectoral engagement was time-consuming and labour-intensive, and effective dissemination to different professional cultures (academia, clinical practice, policy makers, and the community) required the adaptation of research evidence in order to remain relevant to the diverse audiences. Specifically in targeting the general population using national television as a channel of communication to stimulate and initiate fruitful discussions on the issue, it was imperative to adapt the research findings, in this case through the use of the “docu-drama”. This model of adaptation of research evidence better appealed to the target audience.

Bringing together decision makers who can engage with one another and use research evidence in policy formulation is, in our experience, the most important predictor for seeing the findings applied. The positionality of the RDD (as a research organisation nested within the government infrastructure), meant that it was able to make links between research and policy and bring together relevant government departments and researchers to ensure that research evidence is digested and responded to. HIV pushes us to think in new ways and create broad partnerships between disciplines and government departments. The effect of HIV on OVC, in the case of Ghana, dictated that representatives from several sectors collaborated to create an impact that would not have been possible with a single institution; the existence of an autonomous institution embedded within the government mechanism can create a vital link between those sectors.

## Abbreviations

ABBA: Addressing the Balance of Burdens of HIV/AIDS; DFID: Department for International Development; DSW: Department of Social Welfare; GAC: Ghana AIDS Commission; GHS: Ghana Health Service; MOWAC: Ministry of Women and Children Affairs; OVC: Orphans and Vulnerable Children; PLHA: Persons Living With HIV/AIDS; RDD: Research and Development Division; RPC: Research Program Consortium

## Competing interests

This paper critically reflects on a research project in which the authors were involved.

## Authors' contributions

JOG drafted the paper, RAS and KAA contributed to drafting and revising the manuscript. All authors read and approved the final manuscript.
